# Advancing medical AI: GPT-4 and GPT-4o surpass GPT-3.5 in Taiwanese medical licensing exams

**DOI:** 10.1371/journal.pone.0324841

**Published:** 2025-06-04

**Authors:** Yao-Cheng Wu, Yun-Chi Wu, Ya-Chuan Chang, Chia-Ying Yu, Chun-Lin Wu, Wen-Wei Sung

**Affiliations:** 1 School of Medicine, Chung Shan Medical University, Taichung, Taiwan; 2 Department of Urology, Chung Shan Medical University Hospital, Taichung, Taiwan; 3 Institute of Medicine, Chung Shan Medical University, Taichung, Taiwan; Philadelphia University, JORDAN

## Abstract

**Background:**

Chat Generative Pre-Trained Transformer (ChatGPT), launched by OpenAI in November 2022, features advanced large language models optimized for dialog. However, the performance differences between GPT-3.5, GPT-4, and GPT-4o in medical contexts remain unclear.

**Objective:**

This study evaluates the accuracy of GPT-3.5, GPT-4, and GPT-4o across various medical subjects. GPT-4o’s performances in Chinese and English were also analyzed.

**Methods:**

We retrospectively compared GPT-3.5, GPT-4, and GPT-4o in Stage 1 of the Taiwanese Senior Professional and Technical Examinations for Medical Doctors (SPTEMD) from July 2021 to February 2024, excluding image-based questions.

**Results:**

The overall accuracy rates of GPT-3.5, GPT-4, and GPT-4o were 65.74% (781/1188), 95.71% (1137/1188), and 96.72% (1149/1188), respectively. GPT-4 and GPT-4o outperformed GPT-3.5 across all subjects. Statistical analysis revealed a significant difference between GPT-3.5 and the other models (p < 0.05) but no significant difference between GPT-4 and GPT-4o. Among subjects, physiology had a significantly higher error rate (p < 0.05) than the overall average across all three models. GPT-4o’s accuracy rates in Chinese (98.14%) and English (98.48%) did not differ significantly.

**Conclusions:**

GPT-4 and GPT-4o exceed the accuracy threshold for Taiwanese SPTEMD, demonstrating advancements in contextual comprehension and reasoning. Future research should focus on responsible integration into medical training and assessment.

## Introduction

Recently, artificial intelligence (AI) has attracted worldwide attention for its rapid development, affecting numerous fields, such as education, transportation, manufacturing, and customer service. Its potential impact on healthcare is particularly compelling. Chat Generative Pre-Trained Transformer (ChatGPT), an AI language processing model, was introduced by OpenAI in November 2022. As one of the most powerful large language models (LLMs), ChatGPT has sparked a revolution across various domains due to its advanced deep learning technique. With its sophisticated language processing and generative abilities, it rapidly reached 100 million users within two months.

Previously, AI models faced challenges in the medical domain, struggling with natural language understanding, the complexity of medical information, accuracy in answering questions, and resource constraints. Several studies have assessed whether GPT-3.5 could pass medical licensing exams, with some reporting unsuccessful outcomes [1,2]. However, after the release of GPT-4 in 2023, expectations for AI applications in healthcare increased. Advances in GPT-4, including its image-processing capabilities and multifunctionality, have suggested potential applications in the medical field. As a result, research on GPT-4’s performance in medical certification exams has expanded, demonstrating notable improvements in accuracy [3–5]. Subsequently, GPT-4o was launched in May 2024, featuring enhanced visual and audio comprehension, various input/output modalities, faster response times, and improved multilingual capabilities. Nevertheless, its application in the medical field remains underexplored.

To assess ChatGPT’s future role in medicine, many studies have attempted to assess its performance in answering medical questions across various medical domains. ChatGPT’s accuracy has varied across specialties, including internal medicine [6], obstetrics and gynecology [7], radiology [8], and orthopedics [9]. Despite these variations, performance has generally remained high across different question types and levels of difficulty.

However, most existing studies have been conducted in English, with limited investigation into ChatGPT’s performance in other languages. A few studies have evaluated ChatGPT in Chinese, such as one confirming that GPT-3.5 failed to pass the Taiwanese Family Medicine Board Exam [2] and another demonstrating that GPT-4 can pass Stage 1 of the two-stage Taiwanese Senior Professional and Technical Examinations for Medical Doctors (SPTEMD) [10]. However, direct comparisons among GPT-3.5, GPT-4, and GPT-4o in Chinese remain scarce. This gap underscores the need for further research to provide a more comprehensive understanding of ChatGPT’s performance across languages.

In this article, we employed a retrospective comparative approach to evaluate ChatGPT’s performance in Stage 1 of the Taiwanese SPTEMD, a Chinese-language medical licensing exam that assesses medical graduates’ competence. We also analyzed the accuracy of GPT-4o in both Chinese and English.

## Methodology

### ChatGPT

ChatGPT was first launched by OpenAI in November 2022. It is an LLM and a neural-network, machine-learning model with over tens of billions of parameters, allowing it to use natural language processing to perform human-like summarization. The initial model, GPT-3.5, features a staggering 175 billion parameters and is freely accessible to all users. GPT-4, which was released in March 2023, was subsequently updated in September 2023 to allow ChatGPT to recognize images. The advanced model, GPT-4o, was introduced in May 2024 and features diverse input/output modalities, including images, audio, and text.

### Data sources

SPTEMD is an examination for Taiwanese medical students, and only those who pass the exam can obtain a Taiwanese medical license. The SPTEMD consists of two stages; both are held twice a year and are organized by the Taiwanese government’s Ministry of Examination. Undergraduate medical students must complete their basic medical studies and achieve passing grades to be eligible for the first stage of the examination. In the second stage, medical students must have completed their clerkship and obtained their graduation certificates. Only after passing the Stage 1 SPTEMD are medical students eligible to take the second examination.

The Stage 1 SPTEMD spans a duration of four hours, divided into two sessions, Medicine Ⅰ and Medicine Ⅱ, each consisting of 100 multiple-choice questions. All questions have a description alongside four options, with only one option being correct. However, sometimes there may be situations where multiple options are considered correct due to ambiguities in the exam questions, which are subsequently corrected by the Ministry of Examination. The set of 200 questions is divided among 10 subjects: In the group “Medicine Ⅰ” are anatomy, embryology, histology, microbiology and immunology, human parasitology, and public health. In the group “Medicine Ⅱ” are physiology, biochemistry, pharmacology, and pathology. There is an uneven distribution in the number of questions about each subject. The questions are predominantly written in traditional Chinese but contain numerous English medical terms.

### Study design

To examine accuracy patterns in GPT-3.5, GPT-4, and GPT-4o across medical subjects, we analyzed questions from Stage 1 of the Taiwanese SPTEMD administered between July 2021 and February 2024. All exam questions were publicly accessible through the Ministry of Examination’s website. The distribution of the subjects is presented in Table 1, which remained unchanged from July 2021 to February 2024. We adopted a retrospective comparative approach, utilizing a standardized dataset of 1,200 multiple-choice questions, ensuring a systematic and reproducible evaluation of GPT models over time.

**Table 1:** Summary of the scores of GPT-3.5, GPT-4, and GPT-4o in Stage 1 SPTEMD.

Subject	Amount	Feb 2024				Jul 2023				Feb 2023				Jul 2022				Feb 2022				Jul 2021			
		n	GPT3.5	GPT4	GPT4o	n	GPT3.5	GPT4	GPT4o	n	GPT3.5	GPT4	GPT4o	n	GPT3.5	GPT4	GPT4o	n	GPT3.5	GPT4	GPT4o	n	GPT3.5	GPT4	GPT4o
Anatomy	31	31	17	27	30	31	21	30	31	31	20	29	30	29	17	28	28	30	18	29	29	31	14	30	31
Embryology^a^	5	5	4	5	5	5	2	5	5	5	3	5	5	5	2	5	5	5	4	5	5	5	4	5	5
Histology	10	9	6	8	9	10	6	10	10	10	7	10	10	10	7	10	10	10	4	9	10	10	7	10	10
Physiology	27	27	13	25	25	27	15	24	24	27	17	25	24	27	15	25	25	27	16	25	25	27	14	25	27
Biochemistry^b^	27	27	20	26	27	27	17	27	27	27	22	27	25	27	19	25	26	27	18	27	26	27	20	27	26
Microbiology^c^	28	28	20	28	28	28	22	27	28	28	21	27	27	28	21	28	28	28	21	26	28	28	23	28	28
Parasitology	7	7	1	6	6	7	4	7	7	7	5	7	7	7	3	5	6	7	5	6	5	7	4	7	7
Public health	15	15	10	13	14	15	14	15	15	15	9	13	13	15	7	15	12	15	12	15	15	15	6	13	10
Pharmacology	25	25	20	24	25	25	18	23	25	25	16	24	25	25	20	25	25	25	17	25	25	25	14	25	24
Pathology	25	23	20	23	23	25	17	24	25	25	18	24	25	22	12	19	22	24	16	24	23	23	16	23	23
Total	200	197	131	185	192	200	136	192	197	200	138	191	191	195	123	185	187	198	131	191	191	198	122	193	191

n, the number of questions remaining after the exclusion of image-based questions; GPT3.5, the number of questions correctly answered by GPT-3.5; GPT4, the number of questions correctly answered by GPT-4; GPT4o, the number of questions correctly answered by GPT-4o; ^a^Embryology and Development Biology; ^b^Biochemistry and Molecular Biology; ^c^Microbiology and Immunology.

**Fig 1:**
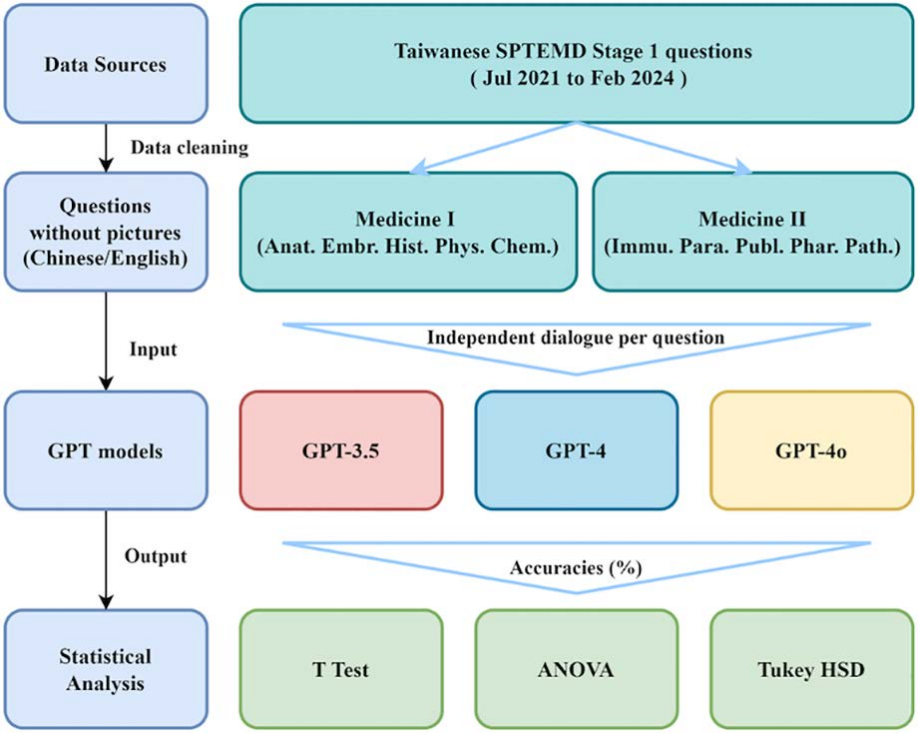
Flowchart for evaluating ChatGPT’s performance in Taiwanese Senior Professional and Technical Examinations for Medical Doctors.

To maintain standardized testing conditions and prevent contextual learning, each question was manually input into the interfaces of GPT-3.5, GPT-4, and GPT-4o as a separate query (Fig 1). Since GPT-4 and GPT-4o support image processing but GPT-3.5 does not, all image-based questions were excluded to ensure consistency across models (n = 1,188 after excluding image-based questions). Additionally, we applied standardized formatting to all questions and randomized their order to minimize bias. Model-generated responses were then compared with official answers provided by the Ministry of Examination. Accuracy rates were calculated and analyzed across subjects to assess the performance differences among the models.

### Study analysis

Statistical analyses were conducted using Microsoft Excel (Microsoft, Redmond, WA, USA) and Python (Python Software Foundation, Wilmington, DE, USA). The accuracy data for GPT-3.5, GPT-4, and GPT-4o were compared using a one-way analysis of variance (ANOVA) and Student’s t-test to identify any significant differences in model performance. Tukey’s honest significant difference (HSD) test was applied as a post hoc analysis to determine pairwise comparisons between the models. Statistical significance was set at p < 0.05 for all analyses.

## Result

### GPT-4 and GPT-4o achieve higher total scores in Medicine I and Medicine II

The results of GPT-3.5, GPT-4, and GPT-4o on answering Medicine Ⅰ and Medicine Ⅱ of the Stage 1 Taiwanese SPTEMD from July 2021 to February 2024 are shown in Table 1. Questions that contained image information were excluded. The assessment was conducted using 1,188 questions for GPT-3.5, GPT-4, and GPT-4o.

**Fig 2:**
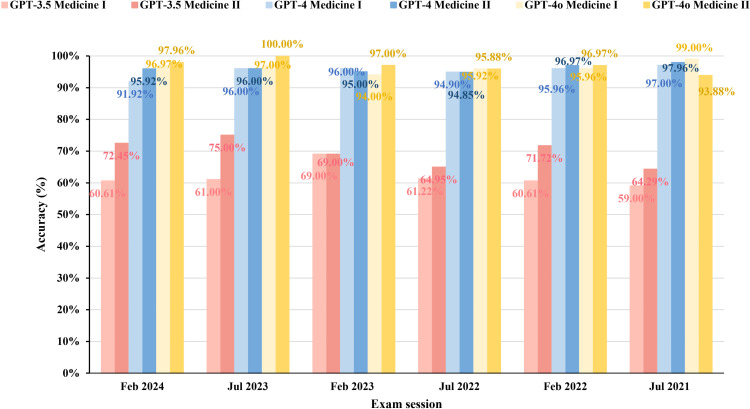
Accuracy of GPT-3.5, GPT-4, and GPT-4o in responding to the Taiwanese Senior Professional and Technical Examinations for Medical Doctors, July 2021 to February 2024.

**Fig 3:**
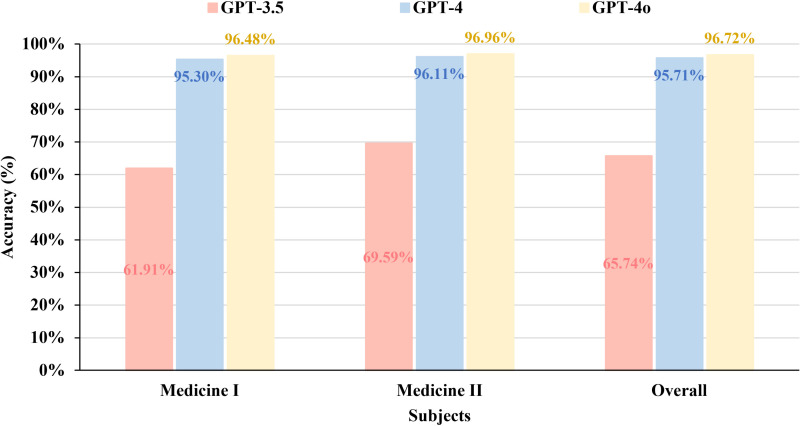
The overall accuracy of GPT-3.5, GPT-4, and GPT-4o in Medicine I, Medicine II, and the combined Medicine I and Medicine II.

The accuracy of the three models in answering different exam sets is documented in Fig 2. The overall accuracy of GPT-3.5, GPT-4, and GPT-4o was 65.74% (781/1188), 95.71% (1137/1188), and 96.72% (1149/1188), respectively (Fig 3). For Medicine Ⅰ, the overall accuracy of GPT-3.5, GPT-4, and GPT-4o was 61.91% (369/596), 95.30% (568/596), and 96.48% (575/596), respectively. For Medicine Ⅱ, the overall accuracy of GPT-3.5, GPT-4, and GPT-4o was 69.59% (412/592), 96.11% (569/592), and 96.96% (574/592), respectively.

### Exploring the performance differences of GPT models across various subjects

Table 2 presents the performance of GPT-3.5, GPT-4, and GPT-4o across different subjects in Stage 1 Taiwanese SPTEMD. Accuracy ranged from 52.38% to 76.19% for GPT-3.5, from 90.48% to 100.00% for GPT-4, and from 87.78% to 100.00% for GPT-4o. The subjects with the highest accuracy for GPT-3.5 were microbiology and immunology (76.19%), followed by biochemistry (71.60%), pharmacology (70.00%), pathology (69.72%), public health (64.44%), embryology (63.33%), histology (62.71%), anatomy (58.47%), physiology (55.56%), and human parasitology (52.38%). For GPT-4, the highest accuracy was in embryology (100.00%), followed by biochemistry (98.15%), microbiology and immunology (97.62%), pharmacology (97.33%), histology (96.61%), pathology (96.48%), anatomy (94.54%), public health (93.33%), physiology (91.98%), and human parasitology (90.48%). In the case of GPT-4o, embryology (100.00%) and histology (100.00%) ranked highest, with microbiology and immunology (99.40%), pharmacology (99.33%), pathology (99.30%), biochemistry (96.91%), anatomy (96.72%), physiology (92.59%), human parasitology (90.48%), and public health (87.78%) following closely.

**Table 2:** Performance of GPT-3.5, GPT-4, and GPT-4o according to different subjects.

Subject	Accuracy (%)		
	GPT-3.5	GPT-4	GPT-4o
Anatomy	58.47	94.54	97.81
Embryology^a^	63.33	100.00	100.00
Histology	62.71	96.61	100.00
Physiology	55.56	91.98	92.59
Biochemistry^b^	71.60	98.15	96.91
Microbiology^c^	76.19	97.62	99.40
Parasitology	52.38	90.48	90.48
Public health	64.44	93.33	87.78
Pharmacology	70.00	97.33	99.33
Pathology	69.72	96.48	99.30
Total	65.75	95.71	96.72

^a^Embryology and Development Biology; ^b^Biochemistry and Molecular Biology; ^c^Microbiology and Immunology.

**Fig 4:**
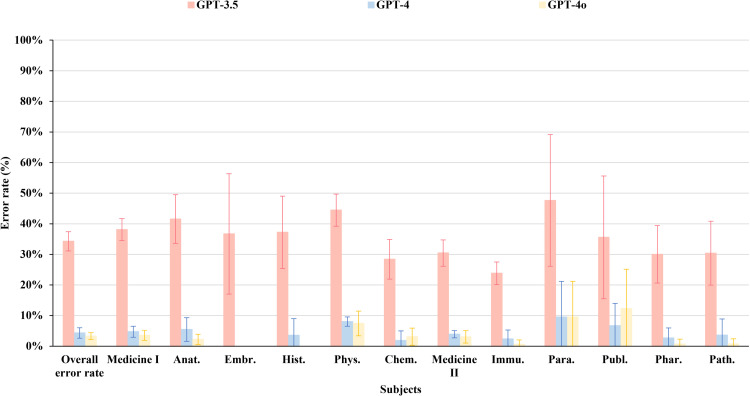
Average error rate across various subjects. Data are presented as mean ± SD.

Fig 4 presents the average error rates of GPT-3.5, GPT-4, and GPT-4o across the subjects. To evaluate performance differences, Student’s t-test was employed to compare the subject-specific error rate against each model’s overall average error rate. The analysis revealed that physiology scored a significantly higher error rate (p < 0.05) than the average for all three models. In GPT-3.5, microbiology and immunology exhibited a significantly lower error rate (p < 0.05) compared to its overall average. For GPT-4, embryology demonstrated a significantly lower error rate than the model’s average (p < 0.05). Meanwhile, GPT-4o displayed a significantly lower error rate in embryology, histology, microbiology and immunology, pharmacology, and pathology compared to its overall average (p < 0.05).

### Comparative accuracy performance of GPT-3.5, GPT-4, and GPT-4o

**Fig 5:**
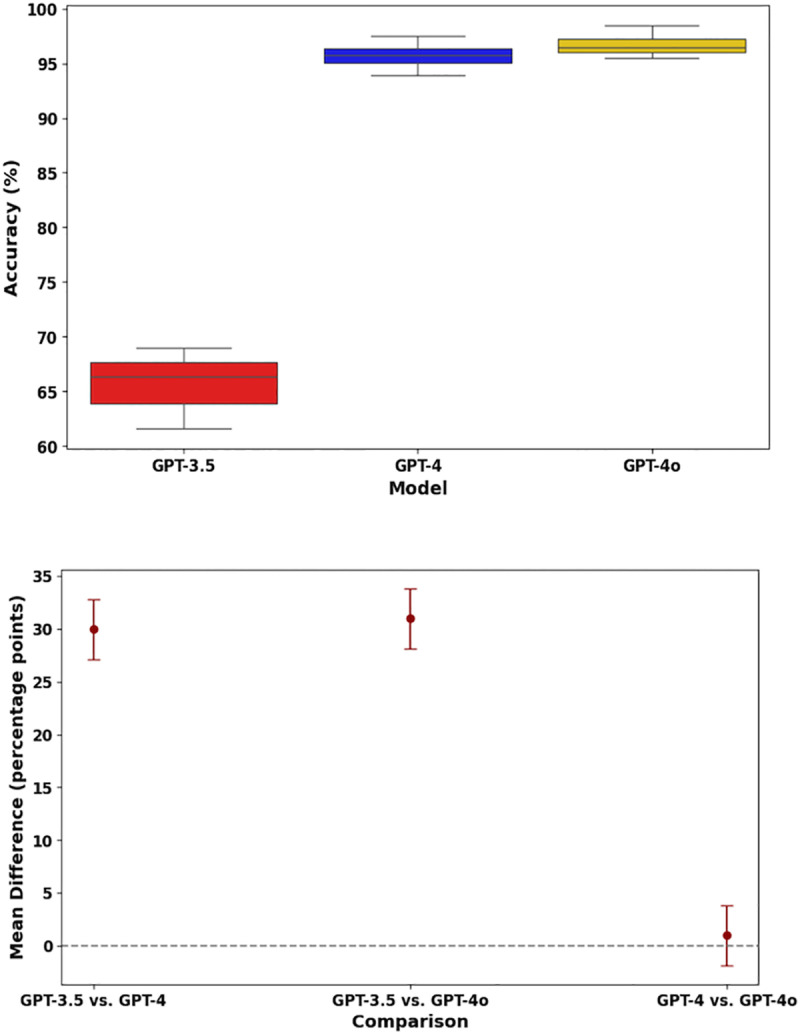
(A) Distribution of accuracy for GPT models and (B) Tukey’s HSD test results. Data are presented as mean ± 2SD.

Fig 5A illustrates the comparative performance of GPT-3.5, GPT-4, and GPT-4o in terms of accuracy, as analyzed using ANOVA, with the box plot showing model types on the X-axis and accuracy on the Y-axis. The box plot clearly shows a significant upward shift in accuracy from GPT-3.5 to GPT-4 and GPT-4o. GPT-3.5 had the lowest median accuracy (0.65) with a wider interquartile range, indicating greater variability in its performance across the tests. In contrast, both GPT-4 and GPT-4o exhibited significantly higher median accuracies (0.96 and 0.97, respectively) and a more consistent performance, reflected in their narrower interquartile ranges. While Tukey’s post hoc analysis (Fig 5B) revealed a significant difference between GPT-3.5 and the other models (p < 0.05), no significant difference was observed between GPT-4 and GPT-4o.

### Investigating the impact of language translation in GPT-4o

To investigate potential differences in GPT-4o’s performance between Chinese and English, we translated the Stage 1 Taiwanese SPTEMD questions into English using GPT-4o and input the English versions into the GPT-4o interface. The results were collected and compared across both language versions. The accuracy rates showed no significant difference, with the Chinese responses achieving 98.14% accuracy and the English responses achieving 98.48% accuracy.

## Discussion

Previous studies have demonstrated that GPT-4 can pass medical licensing exams from various countries; however, data on GPT-4o and its applicability in Chinese-language medical contexts remain unexplored. This is the first study to analyze trends in the accuracy of GPT-3.5, GPT-4, and GPT-4o across different subjects in Stage 1 SPTEMD in Taiwan, providing critical insights into GPT-4o’s performance in Chinese medical assessments. The findings demonstrate that GPT-4 and GPT-4o outperform GPT-3.5 in overall accuracy (95.71% and 96.72%, respectively, vs. 65.74%), as well as in the accuracy of Medicine Ⅰ (95.30% and 96.48%, respectively, vs. 61.91%) and Medicine Ⅱ (96.11% and 96.96%, respectively, vs. 69.59%), significantly exceeding the 60% threshold. Moreover, GPT-4 and GPT-4o maintained over 90% accuracy in all subjects except public health for GPT-4o (87.78%). Statistically, ANOVA and Tukey’s post hoc analysis confirmed that GPT-4 and GPT-4o offer superior accuracy compared to GPT-3.5 (p < 0.05). Both models displayed comparable performance, with no significant difference between them. These findings highlight GPT-4o’s strong capability in Chinese medical contexts, paving the way for future AI-driven advancements in medical education and clinical decision-making.

The highest accuracy for GPT-4 was observed in embryology (100.00%), while GPT-4o excelled in both embryology (100.00%) and histology (100.00%). In contrast, GPT-3.5 performed best in microbiology and immunology (76.19%). Notably, embryology achieved significantly higher accuracy in both GPT-4 and GPT-4o, demonstrating exceptional performance in this subject area. Conversely, the subjects with the lowest accuracy included human parasitology for GPT-3.5 (52.38%), human parasitology for GPT-4 (90.48%), and public health for GPT-4o (87.78%). Among all the subjects, only physiology exhibited significantly lower performance across all the models (p < 0.05), with accuracy rates of 55.56% for GPT-3.5, 91.98% for GPT-4, and 92.59% for GPT-4o. This underperformance in physiology may be attributed to the increased word count in some physiology questions, potentially affecting the comprehension abilities of the GPT models due to the length of the input. Nevertheless, given the variability across subjects and the limited sample size, further studies are needed to confirm these findings and better understand the factors influencing performance disparities.

The influence of language on AI model performance in the medical domain remains an underexplored area of research. Our results showed a slight improvement in performance when using English (98.48%) compared to Chinese (98.14%), although the difference was not statistically significant. This suggests that GPT-4o can reliably process and respond to Stage 1 SPTEMD questions in both languages with near-equivalent accuracy. Given its high performance across both linguistic contexts, GPT-4o could serve as a valuable tool for multilingual medical training and examination preparation. Future research should further investigate the nuances of AI language processing in medical applications and explore how linguistic variations might impact AI-driven learning and clinical decision support.

Since GPT-3.5 was launched by OpenAI in November 2022, a growing number of studies have striven to examine GPT-3.5’s ability to correctly answer questions in medical-licensing exams. Gilson et al. [1] first evaluated the performance of GPT-3.5 on Step 1 and Step 2 United States Medical Licensing Examination (USMLE) exams. Four datasets were analyzed: AMBOSS-Step1, AMBOSS-Step2, NBME-Free-Step1, and NBME-Free-Step2, yielding accuracy of 44%, 42%, 64.4%, and 57.8%, respectively, with only one exceeding the 60% passing threshold. Despite these modest results, GPT-3.5 demonstrated the capacity to provide logical and informative explanations. Similar findings were observed in Taiwanese licensing exams, where GPT-3.5 struggled with the traditional Chinese format of the Family Medicine Board Exam, achieving an accuracy of just 41.6% [2]. A comparable trend emerged in the Taiwanese pharmacist licensing exam, with accuracy rates of 54.4% and 53.8% in the first and second stages for traditional Chinese questions, respectively, improving to 56.9% and 67.6%, respectively, for English-based questions [11]. However, in the Taiwanese Registered Nurse License Exam, GPT-3.5 managed to achieve passing scores in some instances, with accuracy ranging from 54.05% to 63.75% [12].

In contrast, GPT-4 demonstrated markedly improved performance in licensing exams worldwide. In Germany, it achieved 85% accuracy on medical licensing exams, significantly outperforming GPT-3.5’s 58%, particularly excelling in internal medicine and surgery [13]. Similarly, GPT-4 attained an 88.6% accuracy rate on the Saudi Medical Licensing Exam, demonstrating good proficiency for easy and average questions [14]. In the Japanese National Medical Licensing Examination, it surpassed passing thresholds with 82.7% and 77.2% accuracy in essential questions and basic and clinical questions, respectively [15]. In Taiwan, GPT-4 performed well in Stage 1 SPTEMD, achieving an average score of 87.8% across three exam sessions, with subject-specific accuracy ranging from 80.0% in embryology to 93.8% in biochemistry [10]. Advanced medical licensing exams from 2022 to 2023 further confirmed GPT-4’s capabilities, with overall accuracy ranging from 90% to 98% [3]. Comparatively, research on GPT-4o has remained limited since its launch in May 2024. Liu et al. [16] reported that GPT-4o significantly outperformed both GPT-3.5 and GPT-4 in Taiwan’s Emergency Medicine Specialist Examination. However, in our study, no significant difference was found between GPT-4 and GPT-4o, likely due to the foundational medical knowledge of Stage 1 SPTEMD, which contrasts with the more dynamic and evolving field of emergency medicine.

The findings of this study highlight the strengths of GPT-3.5, GPT-4, and GPT-4o in processing multiple-choice medical exam questions. The models demonstrate high accuracy in various subject areas, with noticeable improvements in GPT-4 and GPT-4o over GPT-3.5, reflecting advancements in contextual comprehension and reasoning. Moreover, this study addresses a significant research gap by systematically evaluating the performance of ChatGPT models in both Chinese and English, offering new insights into language-dependent variations in AI-generated responses. Prior research has primarily focused on English-based evaluations, leaving cross-linguistic differences largely unexplored. By analyzing GPT-4o’s performance in two languages, this study provides valuable data for future researchers investigating AI’s role in multilingual medical education and assessment. Additionally, the structured comparison of model versions enhances the understanding of AI-driven reasoning development and contributes to the refinement of AI applications in clinical practice.

The application of AI in medicine requires careful ethical evaluation. This study does not involve real patient data, as all test questions were publicly accessible through the Ministry of Examination. However, the real-world implementation of AI in healthcare must adhere to strict data privacy regulations. Before AI is integrated into clinical workflows, ensuring data anonymization and secure model deployment should be a priority. Future work should focus on developing regulatory frameworks that ensure that AI applications adhere to ethical standards.

Our study has several limitations. First, the lack of performance data from medical students taking the SPTEMD exam prevents direct comparisons between ChatGPT and human test-takers. Second, while this study evaluates AI performance in a standardized exam setting, its applicability to real-world medical practice remains uncertain. Future research should compare AI-generated responses with actual physician decision-making to assess their clinical utility. Third, since GPT models are trained on general internet data, their understanding of region-specific medical guidelines may be limited. Consequently, their generalizability across different medical exams, healthcare systems, and regions remains unclear. Last, while prior studies have highlighted ChatGPT’s interpretability in USMLE assessments [1], concerns persist regarding its transparency due to the proprietary nature of the model and the absence of a publicly available application programming interface. The lack of explainability raises concerns regarding the reliability of AI-generated responses in real-world medical applications.

In summary, this study demonstrates that GPT-4 and GPT-4o consistently surpass the passing threshold of the Taiwanese SPTEMD, whereas GPT-3.5 exhibits variable performance across subjects, with some scores potentially falling below the threshold. The substantial improvements observed in GPT-4 and GPT-4o highlight advancements in contextual comprehension and reasoning, underscoring the rapid evolution of AI in medical knowledge assessment. GPT-4o also excels in Chinese and English, with no significant difference, suggesting that it could serve as a valuable tool for multilingual medical training. While ChatGPT cannot replace clinical decision-making in the near future, it aids in scientific learning, problem-solving, and clinical reasoning through advanced language modeling. Future research should ensure its responsible integration into medical training and assessment.

## References

[1] Gilson A, Safranek CW, Huang T, Socrates V, Chi L, Taylor RA, et al. How Does ChatGPT Perform on the United States Medical Licensing Examination (USMLE)? The Implications of Large Language Models for Medical Education and Knowledge Assessment. JMIR Med Educ. 2023;9:e45312. doi: 10.2196/45312 36753318 PMC9947764

[2] Weng T-L, Wang Y-M, Chang S, Chen T-J, Hwang S-J. ChatGPT failed Taiwan’s Family Medicine Board Exam. J Chin Med Assoc. 2023;86(8):762–6. doi: 10.1097/JCMA.0000000000000946 37294147 PMC12755538

[3] Lin S-Y, Chan PK, Hsu W-H, Kao C-H. Exploring the proficiency of ChatGPT-4: An evaluation of its performance in the Taiwan advanced medical licensing examination. Digit Health. 2024;10:20552076241237678. doi: 10.1177/20552076241237678 38449683 PMC10916498

[4] Yanagita Y, Yokokawa D, Uchida S, Tawara J, Ikusaka M. Accuracy of ChatGPT on Medical Questions in the National Medical Licensing Examination in Japan: Evaluation Study. JMIR Form Res. 2023;7:e48023. doi: 10.2196/48023 37831496 PMC10612006

[5] Wang H, Wu W, Dou Z, He L, Yang L. Performance and exploration of ChatGPT in medical examination, records and education in Chinese: Pave the way for medical AI. Int J Med Inform. 2023;177:105173. doi: 10.1016/j.ijmedinf.2023.105173 37549499

[6] Ayers JW, Poliak A, Dredze M, Leas EC, Zhu Z, Kelley JB, et al. Comparing Physician and Artificial Intelligence Chatbot Responses to Patient Questions Posted to a Public Social Media Forum. JAMA Intern Med. 2023;183(6):589–96. doi: 10.1001/jamainternmed.2023.1838 37115527 PMC10148230

[7] Li SW, Kemp MW, Logan SJS, Dimri PS, Singh N, Mattar CNZ, et al. ChatGPT outscored human candidates in a virtual objective structured clinical examination in obstetrics and gynecology. Am J Obstet Gynecol. 2023;229(2):172.e1–172.e12. doi: 10.1016/j.ajog.2023.04.020 37088277

[8] Bhayana R, Krishna S, Bleakney RR. Performance of ChatGPT on a Radiology Board-style Examination: Insights into Current Strengths and Limitations. Radiology. 2023;307(5):e230582. doi: 10.1148/radiol.230582 37191485

[9] Lum ZC. Can Artificial Intelligence Pass the American Board of Orthopaedic Surgery Examination? Orthopaedic Residents Versus ChatGPT. Clin Orthop Relat Res. 2023;481(8):1623–30. doi: 10.1097/CORR.0000000000002704 37220190 PMC10344569

[10] Huang C-H, Hsiao H-J, Yeh P-C, Wu K-C, Kao C-H. Performance of ChatGPT on Stage 1 of the Taiwanese medical licensing exam. Digit Health. 2024;10:20552076241233144. doi: 10.1177/20552076241233144 38371244 PMC10874144

[11] Wang Y-M, Shen H-W, Chen T-J. Performance of ChatGPT on the pharmacist licensing examination in Taiwan. Journal of the Chinese Medical Association. 2023;86(7):653–8. doi: 10.1097/jcma.0000000000000942 PubMed 37227901 PMC12755457

[12] Huang H. Performance of ChatGPT on Registered Nurse License Exam in Taiwan: A Descriptive Study. Healthcare (Basel). 2023;11(21):2855. doi: 10.3390/healthcare11212855 37958000 PMC10649156

[13] Meyer A, Riese J, Streichert T. Comparison of the Performance of GPT-3.5 and GPT-4 With That of Medical Students on the Written German Medical Licensing Examination: Observational Study. JMIR Med Educ. 2024;10:e50965. doi: 10.2196/50965 38329802 PMC10884900

[14] Aljindan FK, Al Qurashi AA, Albalawi IAS, Alanazi AMM, Aljuhani HAM, Falah Almutairi F, et al. ChatGPT Conquers the Saudi Medical Licensing Exam: Exploring the Accuracy of Artificial Intelligence in Medical Knowledge Assessment and Implications for Modern Medical Education. Cureus. 2023;15(9):e45043. doi: 10.7759/cureus.45043 37829968 PMC10566535

[15] Tanaka Y, Nakata T, Aiga K, Etani T, Muramatsu R, Katagiri S, et al. Performance of Generative Pretrained Transformer on the National Medical Licensing Examination in Japan. PLOS Digit Health. 2024;3(1):e0000433. doi: 10.1371/journal.pdig.0000433 38261580 PMC10805303

[16] Liu C-L, Ho C-T, Wu T-C. Custom GPTs Enhancing Performance and Evidence Compared with GPT-3.5, GPT-4, and GPT-4o? A Study on the Emergency Medicine Specialist Examination. Healthcare (Basel). 2024;12(17):1726. doi: 10.3390/healthcare12171726 39273750 PMC11394718

